# Treating Public Health Dilemma of Gingival Recession by the Dehydrated Amnion Allograft: A 5-Year Longitudinal Study

**DOI:** 10.3389/froh.2020.540211

**Published:** 2020-11-11

**Authors:** Santosh Kumar, Tanvi Hirani, Sujay Shah, Rupal Mehta, Susmita R. Bhakkand, Deepak Shishoo

**Affiliations:** ^1^Department of Periodontology, Karnavati School of Dentistry, Karnavati University, Gandhinagar, India; ^2^Department of Physiology, Karnavati School of Dentistry, Karnavati University, Gandhinagar, India

**Keywords:** allograft, amnion, gingival recession, gingival thickness, mucogingival surgery

## Abstract

**Aim:** This study aimed to evaluate the efficacy of dehydrated amnion allograft with coronally positioned flap procedure in paired Miller's class I recession defects.

**Methods:** A total of 51 subjects were included in the study with bilateral Miller's class I gingival recession defects. In the test group, patients were treated with an amniotic membrane (AM) with a coronally positioned flap, while in the control group, patients were treated with coronally positioned flap alone. Clinical parameters such as recession depth, recession width (RW), probing depth (PD), relative attachment level (RAL), width of keratinized gingiva (WKG), and thickness of keratinized gingiva (TKG) were recorded at baseline and after 5 years of follow-up.

**Result:** The mean baseline recession was 2.95 ± 0.89 in the test group and 2.70 ± 0.85 in the control group, and both were statically non-significant. At the end of 6 months, all the parameters, when compared with the baseline, showed a significant improvement. Intergroup comparison showed the non-significant difference in all settings except the TKG.

**Conclusion:** AM proved to help improve the TKG. This increase in thickness helps in the long-term maintenance of the gingival margin in Miller's class I recession defect.

## Introduction

The gingival recession is one of the most significant public health issues [1]. The term gingival recession is coined to characterize the apical shift of marginal gingiva from its normal position [2]. Unlike other dental anomalies, a gingival recession usually exhibits an esthetic problem, primarily when it affects the anterior region of oral cavity. It is also very often associated with dentinal hypersensitivity, root caries, and cervical abrasion due to the exposure of the root surfaces to the oral environment and an increase in plaque accumulation [3]. The prevalence of gingival recession is different in different parts of the world [4]. In India, the prevalence of gingival recession ranges from 24.29 to 67.23% [5].

Facial esthetics involve the interaction of many elements of which the periodontium serves as a backdrop for the teeth, which determines the environment for esthetic rehabilitation and hence periodontal procedures considered as an essential part of the comprehensive cosmetic treatment plan by identifying the esthetic concerns [6].

In dentistry, it is significant to focus on the appearance of teeth along with the function. Gingival recession is the exposure of the root surface by an apical shift in the position of the gingiva. There are many etiological factors implicated in a gingival recession like faulty tooth brushing techniques, tooth malposition, friction from soft tissues, gingival inflammation, abnormal frenum attachment, and iatrogenic dentistry [7].

At present, gingival reconstruction is an integral part of periodontal science [8]. With the advancement in the techniques, it has now become predictable to perform root coverage procedures and augment ridges and to magnify prosthetic reconstruction. However, the root coverage has low long-term predictability, mostly due to the thin gingival phenotype [9]. The thin gingival phenotype is a delicate, highly scalloped soft tissue and is more prone to recession, bleeding, and inflammation [10]. Clinical enhancement from thin to thick, of the gingival phenotype, helps in better treatment outcomes [11, 12]. The adequate width of attached gingiva is critical in maintaining a healthy periodontium [13, 14]. The proper thickness of keratinized gingiva (TKG) provides a firm and stable base for maintaining good oral hygiene [15]. So, there is a need to have an excellent technique supplemented with perfect material to increase the thickness of gingiva.

There are various techniques available for the treatment of gingival recession [16–20], which include using autogenous free gingival grafts [21], autogenous connective tissue grafts [18], and allograft dermis tissue [22]. Furthermore, the enamel matrix derivative, platelet-rich plasma, and recombinant platelet-derived growth factor can be used as biological mediators along with above techniques for a complete wound healing [23–25].

The coronally advanced flap (CAF) procedure is one of the most predictable techniques and considered a gold standard [26]. The term CAF was introduced by Pini-Prato et al. [27], in 1999. The CAF technique not only helps in achieving root coverage but also helps in maintaining high esthetics [19]. The CAF procedure shows improved root coverage, gain in clinical attachment level (CAL), and increased width of keratinized gingiva (WKG) [28]. It does not need a second surgical site, which is an additional discomfort to the patients, as in free gingival graft and connective tissue graft procedure. The long-term success of CAF lot depends on the type of gingival biotype [29]. A thick gingival biotype is always preferred, but many times we come across a thin biotype [30]. So, we need to devise a technique which is an alternative to the conventional method to increase the thickness of gingiva.

Recently, the amniotic membrane (AM) has been introduced in dentistry. For a long time, it has been used in medicine as skin graft, in treatment of burns, and in ulcerated skin conditions with great success [31]. It consists of a single layer of epithelial cells, thin reticular fibers, a slim, compact layer, and a fibroblast layer. It includes bioactive factors such as laminins, the most prevalent being Laminin-5 [32]. It acts as a barrier to secure fetus from trauma and infection because of the lack of a fetal immune system [33]. It is 0.5 mm in thickness. AM decreases inflammation, lessens the occurrence of adhesions, prevents tissue scarring, and also aids in angiogenesis with wound healing [34]. In the wisdom of the advantages of the above membrane, this long-term study was thus undertaken to prove the efficacy of AM. Due to the high prevalence of gingival recession, we conducted this study. The hypothesis of this study states that there will be a significant coverage in Miller's class I defect by this study procedure.

## Materials and Methods

**Figure 1:**
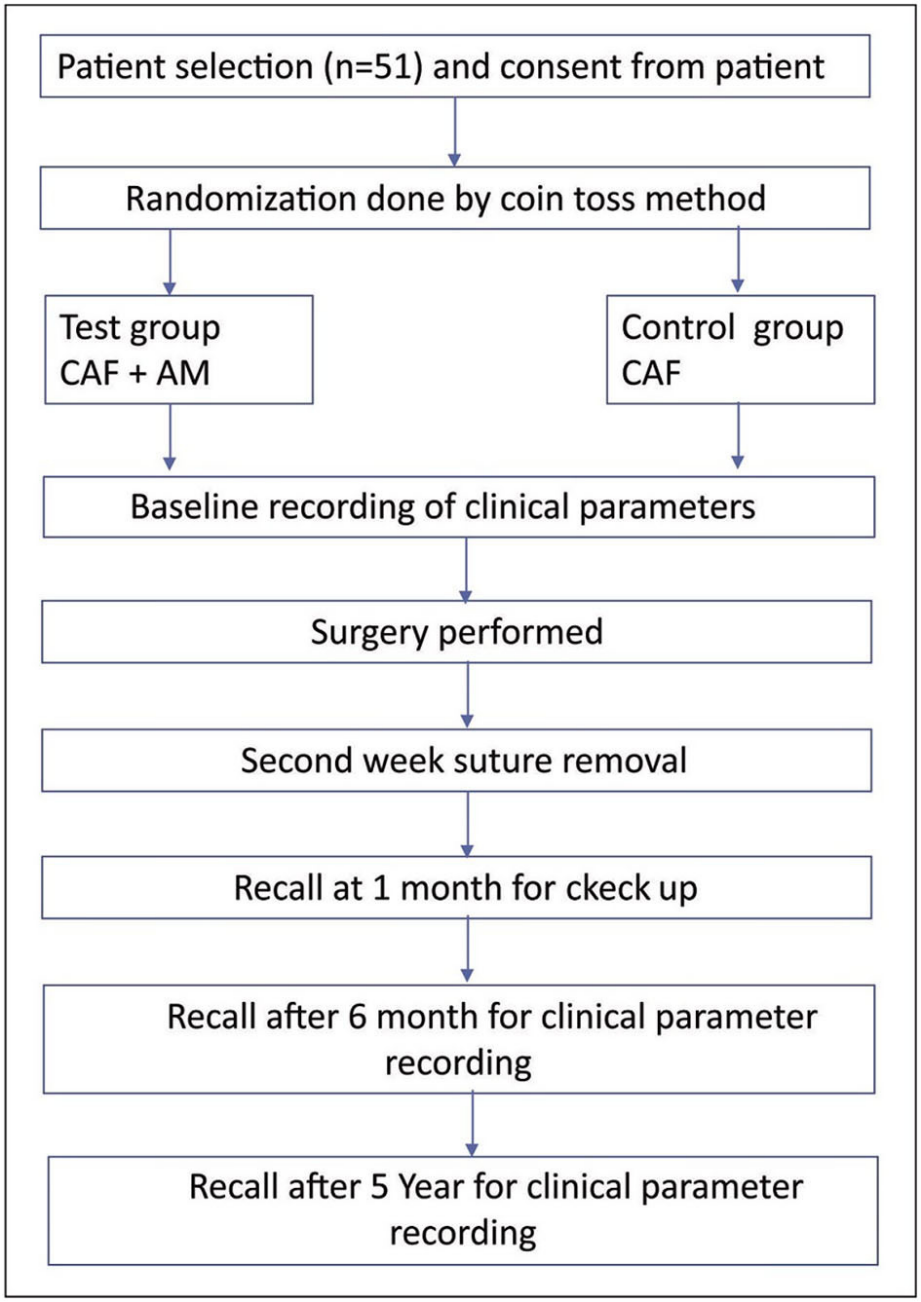
Flowchart for the study showing the methodology.

This comparative study was conducted in the Department of Periodontics. A similar short-term pilot study was conducted in the same department with fewer subjects [12]. Since the earlier study was a short-term study, the samples from the previous study were not included in the current study [12]. Patients were made aware of the study protocol, and written consents were obtained at the beginning of the study. Ethical clearance was obtained from the research and review board committee (KSD/2013/238). This study was conducted in accordance with the declaration of Helsinki. The study procedures were explained as a flowchart (Figure 1). The following formula was used to determine sample size $n={\left(Z_{\alpha /2}+Z_{\beta }\right)}^{2}*2*\sigma^{2}/d^{2}$ (where *Z*_α/2_ is the critical value of the normal distribution at α/2).

### Inclusion Criteria

**Figure 2:**
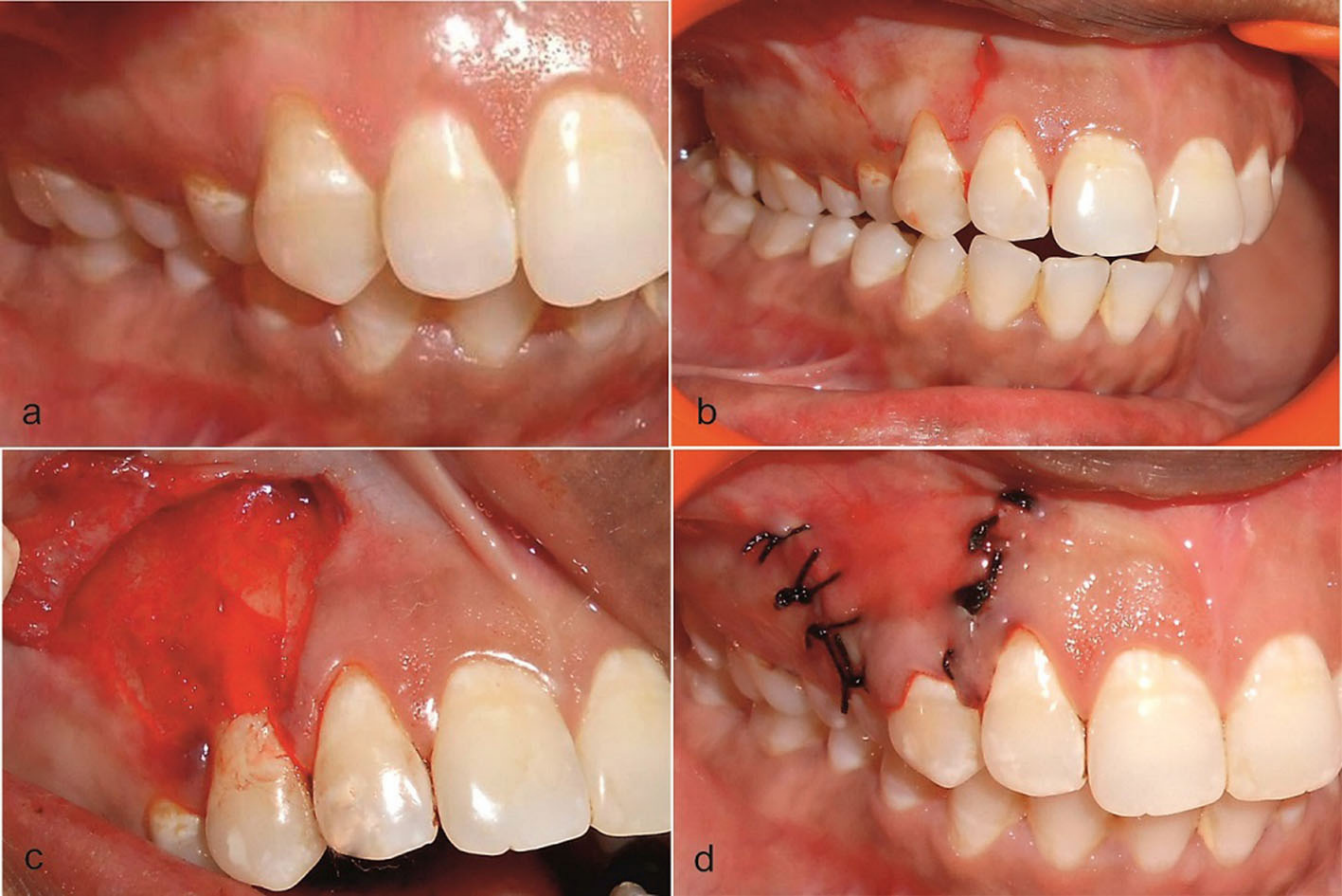
**(a)** Preoperative view of the recession on the upper right first premolar at the test side. **(b)** Incision was placed. **(c)** Trapezoidal mucoperiosteal flap was raised up to the mucogingival junction and AM placed on the recipient site. **(d)** Coronally positioned flap was sutured.

Patients included both males and females, in the age group of 18–40 with presence of isolated bilateral gingival recession in the premolar and anterior regions (Miller's class I recession; Figure 2a), who were systemically and periodontally healthy and had the ability to maintain good oral hygiene and were compliant with all study-related procedures and available for follow up.

### Exclusion Criteria

Patients who had the habit of using tobacco in any form, with malpositioned teeth, and with a history of any previous surgical procedure performed for the correction of recession as well as pregnant and lactating women were excluded from the study.

### Procedure

According to the sample size calculation, 50 patients were required to conduct this study. Considering that it was a 5-year long-term study and anticipating a dropout of 40%, we included 20 patients extra for this study. Hence, the study initially consisted of 70 patients, out of which one passed away due to some medical reasons and 18 patients did not turn up for regular follow-ups. Hence, the remaining 51 patients' data was analyzed. A split-mouth design was applied for this study to minimize the bias, which occurs due to the difference in individual healing factors. Each of the patients was subjected to phase 1 therapy (non-surgical basic periodontal procedure), and later oral hygiene instructions were reinforced. Periodontal evaluations were performed at an interval of a month to access the condition of soft tissue for periodontal surgery. Once the inflammation subsided, root coverage procedures were performed by the single operator. The selected sites were divided into test and control groups by the coin toss method. The allotted test group sites were treated by a CAF along with AM, whereas the control group sites were managed by CAF alone. Patients were not informed about the location of placement of membrane.

### Clinical Parameters

a. Recession depth (RD) consisted of measurements from the mid-buccal region of the gingival margin to the cementoenamel junction (CEJ) and measured by the Williams periodontal probe (Hu-Friedy Mfg. Co, USA).

b. Recession width (RW) measurement was taken at CEJ (mid-buccal level). The periodontal probe was placed horizontally, and mesiodistal distance was measured between marginal gingivas.

c. Probing depth (PD) is the distance from the bottom of the pocket to the free gingival margin and was measured by the periodontal probe.

d. Relative attachment level (RAL).

e. The WKG extends from the mucogingival junction to the deepest part of free gingiva. This was measured by William's periodontal probe.

f. The TKG: The gingival area to be measured was anesthetized using 10% lidocaine spray (Xylonor Spray, Septodont, Saint-Maur-des-Fossés, France). An endodontic Hedstrom file (H file, Number 25) with a rubber stopper was used to measure the gingival thickness. The H file was inserted in the gingiva, perpendicular to the long axis of the tooth, and the rubber stopper was adjusted to contact the surface of the tissue. Once done, the H file was removed from the gingival tissue, and the measurement was taken from the tip of the file to the rubber stopper. These measurements were made 1 mm below the gingival margin. A single examiner (SS) performed all the measurements to minimize the inter-examiner bias [35].

### Surgical Procedure

After measuring all the presurgical parameters, the mouth was rinsed with 0.2% chlorhexidine digluconate, and iodine solution was used extra orally to follow asepsis protocol. The surgical area was anesthetized using 2% lignocaine hydrochloride comprising adrenaline at a concentration of 1:80,000. After obtaining adequate local anesthesia, a horizontal incision was given at the marked level of the CEJ on either side of the tooth involved. Care was taken not to include the marginal gingiva of the adjacent teeth, and an incision was given in a way that preserves the interdental papilla. Two vertical incisions (Figure 2b) extending apically were made slightly divergent, for attaining a broader base for efficient blood supply to the flap section. On the buccal aspect of the involved tooth, an intrasulcular incision was made by using a Bard parker number 15 blade. Both the incisions, intrasulcular and horizontal, were connected, and a full-thickness flap was elevated from the level of the horizontal incision to the mucogingival junction. After reaching the mucogingival junction, this mucoperiosteal flap was split, keeping the periosteum intact. The split-thickness flap was extended till the vestibule, and it was checked if the flap, when pulled coronally, was able to cover the recession without any tension completely. De-epithelialization of interdental papilla was performed to expose the connective tissue. No root biomodification was done [36]. On the test site, AM (Amino-care Biocover Laboratories Pvt Ltd.) was placed under the CAF (Figure 2c). The flap was secured coronally by a sling suture (4–0 black silk suture) (Figure 2d). The periodontal dressing was placed to protect the surgical site. On control sites, all the operative procedures were the same, but for the placement of the AM.

### Clinical Assessments

Customized acrylic stents were prepared for accurate pre and postoperative measurements. The stents were extended till the CEJ apically and one tooth mesially and distally. The stents were placed and stored on the study cast to prevent distortion to a minimal level. Stents were grooved with a thin-tapered bur in the occlusal–apical direction. These grooves act as a standard reference point for the placement of the UNC#15 probe. The below-mentioned clinical parameters were recorded at baseline and after 5 years. Patients were recalled to follow up every 6 months. A different operator who was utterly unaware of the procedures did all the clinical measurements to blind the study.

### Postoperative Care

**Figure 3:**
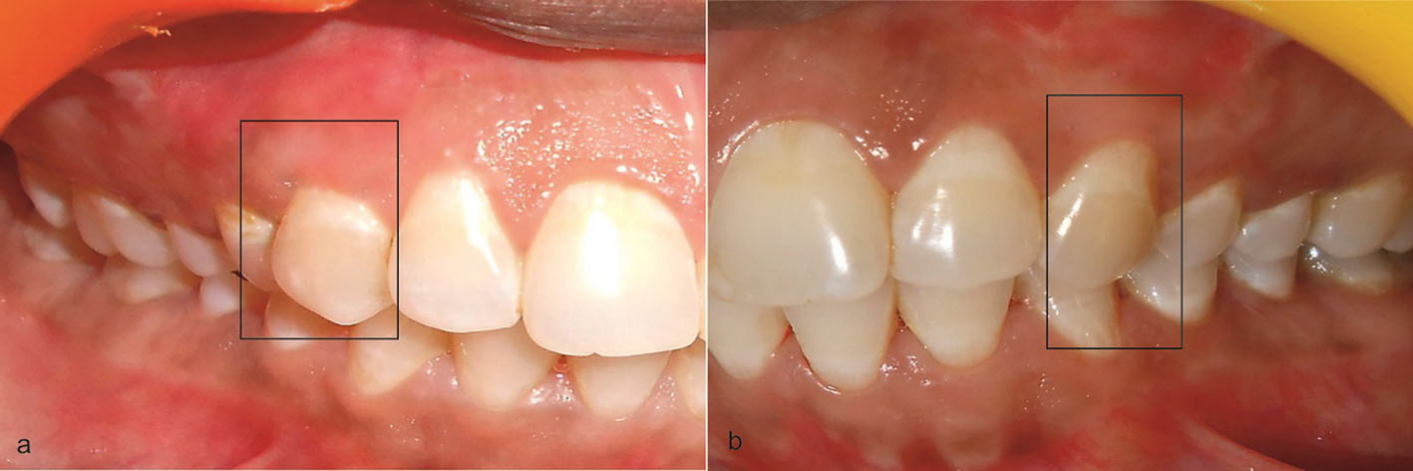
**(a)** Postoperative view at 6 months after initial surgery at test group. **(b)** Postoperative view at 6 months after initial surgery at Control group.

**Figure 4:**
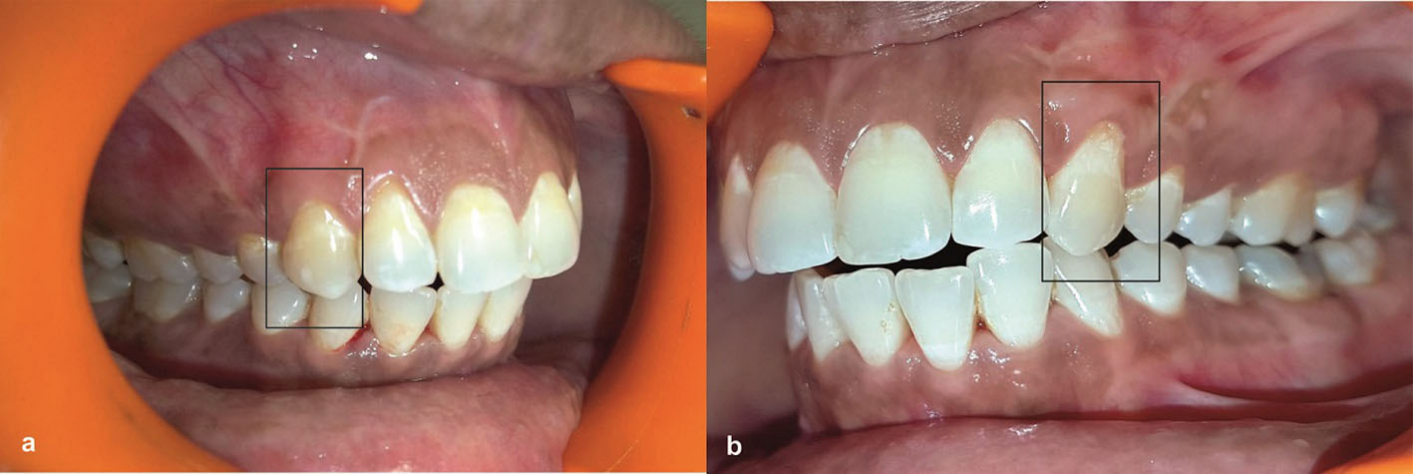
**(a)** Postoperative view at 5 years after initial surgery at test group. **(b)** Postoperative view at 5 years after initial surgery at Control group.

Appropriate antibiotic and analgesic (500 mg amoxicillin, 3 times per day for 5 days, and 50 mg diclofenac sodium, 3 times per day for 3 days) were prescribed in combination with 0.2% chlorhexidine rinse twice daily for 2 weeks. Sutures and periodontal pack (Coe-Pak, Ward's Wondrpak, and Peripac) were removed after 2 weeks postoperatively, and the surgical sites were gently irrigated with 0.2% chlorhexidine digluconate. Later, patients were instructed to brush on surgical sites gently. The patients were clearly instructed for good oral hygiene practices. Each patient was scheduled for recall visit on the 15th day, 1 month, 6 months (Figures 3a,b), 1 year, and 5 years (Figures 4a,b). All the clinical parameters (plaque index, modified gingival index, recession depth, RW, probing pocket depth, WKG, gingival/mucosal thickness) were measured at 6 months and 5 years. The plaque index and modified gingival index were also recorded at baseline. Supportive periodontal therapy was rendered to all the patients until 5 years.

### Statistics

The data were recorded as mean ± standard deviation. The paired *t*-test was applied for intragroup and intergroup comparisons at 6 months and 5 years, and the level of significance of 0.05 was marked for all statistical observations.

## Results

The study consisted of 51 patients with a mean age group of 35.6 years at baseline. At baseline, all the parameters (RD, RW, PD, CAL, WKG, and TKG) corresponding to the test and control group were statically non-significant (Table 1).

**Table 1:** Intergroup comparison of RD, RW, PD, CAL, WKG, and TKG (mm) at baseline, 6 months, and 5 years.

		**Test**	**Control**	***p*-Value**
RD (Mean ± SD)	Baseline	2.95 ± 0.89	2.70 ± 0.85	0.150
	6 Months	0.43 ± 0.45	0.50 ± 0.45	0.454
	5 Years	0.70 ± 0.49	1.7 ± 0.45	0.0001^**^
RW (Mean ± SD)	Baseline	3.10 ± 0.41	3.20 ± 0.79	0.424
	6 Months	0.49 ± 0.50	0.55 ± 0.47	0.533
	5 Years	0.80 ± 0.50	1.12 ± 0.47	0.0012^*^
PD (Mean ± SD)	Baseline	1.20 ± 0.49	1.20 ± 0.45	1.000
	6 Months	1.05 ± 0.12	1.13 ± 0.39	0.164
	5 Years	1.17 ± 0.12	1.20 ± 0.39	0.6007
CAL (Mean ± SD)	Baseline	4.40 ± 1.16	4.10 ± 0.89	0.146
	6 Months	1.53 ± 0.52	1.64 ± 0.50	0.324
	5 Years	2.00 ± 0.52	2.80 ± 0.50	0.0001^**^
WKG (Mean ± SD)	Baseline	3.00 ± 0.75	3.10 ± 0.71	0.490
	6 Months	4.62 ± 0.22	4.62 ± 0.25	0.056
	5 Years	4.00 ± 0.71	3.30 ± 0.69	0.0001^**^
TKG (Mean ± SD)	Baseline	1.77 ± 0.20	1.76 ± 0.25	0.823
	6 Months	2.46 ± 0.22	1.80 ± 0.16	0.0001^**^
	5 Years	2.31 ± 0.22	1.79 ± 0.16	0.0001^**^

* *Significant*.

** *Highly significant*.

Intragroup data after 6 months (Table 2) showed that healing about RD, RW, CAL, and WKG in both the groups was highly significant when compared with baseline. PD measurements in both groups from baseline were statically insignificant. The test group showed a higher gain in the TKG when compared to baseline and was statistically significant (2.46 ± 0.22, *p* = 0.0001), whereas there was a non-significant gain in the control group (1.80 ± 0.16, *p* = 0.3382).

**Table 2:** Intragroup comparison of RD, RW, PD, CAL, WKG, and TKG (mm) at 6 months and 5 years.

**Parameters**	**Test group**	***p*-Value**	**Control group**	***p*-Value**
**RD**
Baseline	2.95 ± 0.89	0.0001^**^	2.70 ± 0.85	0.0001^**^
6 Months	0.43 ± 0.49		0.50 ± 0.45	
Baseline	2.95 ± 0.89	0.001^*^	2.70 ± 0.85	0.001^*^
5 Years	0.70 ± 0.49		1.70 ± 0.45	
**RW**
Baseline	3.10 ± 0.41	0.0001^**^	3.20 ± 0.79	0.0001^**^
6 Months	0.49 ± 0.50		0.55 ± 0.47	
Baseline	3.10 ± 0.41	0.001^*^	3.20 ± 0.79	0.001^*^
5 Years	0.80 ± 0.50		1.12 ± 0.47	
**PD**
Baseline	1.20 ± 0.49	0.240	1.20 ± 0.45	0.070
6 Months	1.10 ± 0.12		1.13 ± 0.39	
Baseline	1.20 ± 0.49	0.675	1.20 ± 0.45	0.905
5 Years	1.17 ± 0.12		1.20 ± 0.39	
**CAL**
Baseline	4.40 ± 1.16	0.0001^*^	4.10 ± 0.89	0.0001^*^
6 Months	1.53 ± 0.52		1.64 ± 0.50	
Baseline	4.40 ± 1.16	0.001^*^	4.10 ± 0.89	0.001^*^
5 Years	2.00 ± 0.52		2.80 ± 0.50	
**WKG**
Baseline	3.00 ± 0.75	0.0001^**^	3.10 ± 0.71	0.0001^**^
6 Months	4.71 ± 0.22		4.62 ± 0.25	
Baseline	3.00 ± 0.75	0.001^*^	3.10 ± 0.71	0.001^*^
5 Years	4.00 ± 0.71		3.30 ± 0.69	
**TKG**
Baseline	1.77 ± 0.20	0.0001^**^	1.76 ± 0.25	0.3382
6 Months	2.46 ± 0.22		1.80 ± 0.16	
Baseline	1.77 ± 0.20	0.0001^**^	1.76 ± 0.25	0.472
5 Years	2.31 ± 0.22		1.79 ± 0.16	

* *Significant*.

** *Highly significant*.

Intergroup comparison after 6 months showed statistically non-significant changes in RD, RW, PD, CAL, and WKG. The gain in the TKG in the test group was more when compared with the control group. Gain TKG was highly significant (*p* = 0.0001) when compared to its control part (Table 1).

Intragroup data after 5 years (Table 2) showed that healing of RD, RW, CAL, and WKG in both the groups was highly significant when compared from baseline whereas change in PD was insignificant from baseline in both the groups (test group: *p* = 0.675, control group: *p* = 0.905). In the test group, gain in the TKG was highly significant (2.31 ± 0.22, *p* = 0.0001) while it was non-significant in the control group (1.79 ± 0.16, *p* = 0.4721).

Intergroup comparison after 5 years showed a statistically significant gain in the test group of TKG (*p* = 0.0001). RD, RW, and CAL showed significant test group gain in all the parameters when compared with the control group (Table 1). At the same time, PD remained non-significant (*p* = 0.6007).

## Discussion

Mucogingival problems are related to many conditions that affect many numbers of individuals. The basic objective of mucogingival corrective surgery is complete coverage of exposed root surfaces and at the same time achievement of good esthetics and function. Gingival recession is one of the most common problems encountered by a periodontist in his/her clinical practice. A variety of factors that lead to the gingival recession are traumatic tooth brushing, malpositioning of teeth, high frenum attachment, alveolar bone dehiscence, plaque, and calculus and iatrogenic factors related to restorative dentistry [2, 4, 37–40]. Apart from the concern for esthetics, recession leads to exposed roots and in turn causes hypersensitivity. This leads to avoidance of plaque control methods at concerned sites. Hence, it initiates the periodontal disease. So, getting maximum coverage is of immense importance.

To obtain maximum coverage, periodontists have vast modalities of treatment options. These techniques include pedicle soft tissue grafts like rotational flaps and advanced flaps [22], free soft tissue grafts [6], and other treatments like root surface modification agents, guided tissue regeneration [19, 20], and enamel matrix proteins. We have to choose the appropriate material to gain the maximum result from the vast amount of options available in today's healthcare industry. Recently, research on AM has shown a promising result.

AM belongs to the innermost lining of the fetal membrane, which safeguards the developing fetus. This membrane acts as a barrier to secure the fetus from trauma and infection because of the lack of the fetal immune system. AM can decrease inflammation, reduce the occurrence of adhesions, prevent scarring of tissue, promote angiogenesis, and aid in wound healing [41, 42]. It also preserves a normal epithelial phenotype by promoting epithelialization and also has an antimicrobial property. It also serves as a basement membrane that helps in epithelial cell migration [43], reinforces the adhesion of basal epithelial a cells [44], promotes epithelial differentiation [45], and prevents epithelial apoptosis [46]. AM also contains growth factors, which aid in the formation of granulation tissue by promoting fibroblast growth and neovascularization [47]. Apart from the above, these tissues contain cells that have characteristics similar to stem cells that magnify the resulting outcome [48].

Amnion has the proven capacity to form a primitive physiologic “seal” with the host tissue and helps to prevent bacterial contamination [49]. Considering the above-stated advantages of the membrane, the objective of this study was to clinically evaluate the effectiveness of the dehydrated AM in conjugation with CAF vs. CAF alone in the treatment of Miller's class I or II gingival recession.

A split-mouth design and a surgery by the single operator were done to reduce the bias mainly determined by differences in individual factors. The study compromised 70 patients, out of which 51 turned up for regular visits. The mean average age of the patients was 35.6 years, and they had bilaterally similar Miller's class I gingival recession. At baseline, preoperatively both the groups were compared to find the similarity of the defect (Table 1). The result showed that all the parameters of recession preoperatively were non-significant. The sites were assigned to each group by the coin toss method. At experimental sites, root coverage was done with CAF and AM whereas at the control sites the coverage was achieved with CAF alone. It was observed that both the methods were almost equally capable of potent root coverage. Results were analyzed first at 6 months. There was a statistically significant reduction in the parameters in both the groups except TKG (Table 2). The percentage of root coverage obtained after 6 months was 85% in the test group and 81% in the control group. The percentage of root coverage in the control group was a little lower than that of the test group but was statistically non-significant (Table 2: *p* = 0.4 the 54), whereas there was a marked improvement in the WKG. In the test group, there was an improvement of 97 and 2.72% in the control group. A minimal current literature that can be compared to this study is available. A study conducted by Brian Gurinsky showed an average increase of 3.2 mm of new gingival tissue which represented 97% of root coverage [50]. In the present study, the mean gain recession of the depth at 6 months is 81%, which may be comparable to the research done by Cordaro et al. [51]. They used a similar technique for CAF and found a mean reduction of 2.29 mm at the end of 6 months. This above result is also identical to the study conducted by Latha et al., which showed a root coverage of 86.86% at the end of 12 months [52].

In the current study, there was a marked increase in the TKG. There was a gain of 97% in the test group and 2.72% in the control group. This study reminds us of a decisive role of the AM. This soft tissue augmentation can result from the proliferation of gingival and periodontal ligament fibroblast arising from the AM. It can also be due to a spacing effect of the membrane. Amnion tissue also contains growth factors that may aid in the formation of granulation tissue by stimulating fibroblast growth and neovascularization [47]. Additionally, the cells found within the tissue exhibit characteristics similar to stem cells [48]. It decreases the host immunological response *via* localized suppression of polymorphonuclear cell migration [7, 53]. It also possesses different varieties of laminin, which are known for cellular adhesion of gingival cells [32]. The beneficial effects of the AM have been studied in various procedures, such as periodontal soft tissue healing [54], periodontal intrabony defects [55], and tissue engineering. Other parameters (RW, PD, CAL, and WKG) in both groups improved considerably at 6 months. Intergroup comparison showed no significant difference (Table 2).

Comparison at 5 years showed significant improvement of RD, RW, CAL, and WKG from the baseline but largely reduced from readings of 6 months in the control group, whereas in the test group reduction from the 6-month level remained very low. The TKG at the test and control sites remained 2.31 ± 0.22 and 1.79 ± 0.16, respectively, at the end of 5 years. If the data is observed carefully, it is clear that at 6 months recession gain was 85% in the test group and 81% in the control group. At the end of 5 years, coverage maintained at 76.2% in the test group and went down to 37% in the control group, which can be attributed in the TKG. This finding is similar to the study done by Irfan et al. [12] which showed root coverage of 86% and thickness increase by 0.74 mm with the use of an AM in combination with that coronally positioned at 6 months.

Olsson and Lindhe. in their study suggested that in 85% of the population, the thick periodontal phenotype was more prevalent than the thin form (15%) [7]. The tissue here is dense and fibrotic with a broad zone of attached gingiva [56]. The other distinctive features of tissue with a thick phenotype include flat soft tissue and bony architecture, denser and more fibrotic soft tissue curtain, a large amount of attached masticatory mucosa, resistance to acute trauma, and hence, less amount of soft tissue loss.

Gingival thickness affects the treatment outcome, probably because of the difference in the amount of blood supply to the underlying bone and susceptibility to resorption [57–59]. Gingival recession is more prone to occur in patients with this phenotype [57]. Tissue phenotype is also an essential factor in determining the esthetic treatment outcome.

Hence, during the planning of treatment, the soft tissue phenotype should be considered as it affects the outcome. Soft tissue contour and thickness are critical diagnostic features. The thick phenotype also shows more excellent dimensional stability in remodeling as compared to the thin phenotype. This may be due to the presence of the laminar bone adjacent to the outer cortical plate, which provides the foundation for metabolic support of the cortical bone and hence its stability and sustainability.

In the present study, the postoperative TKG was the only significant gain which resulted in a gain of other parameters. The soft tissue gain was probably due to the result of the proliferation of gingival and periodontal ligament fibroblast, arising from the AM. It can also be due to a spacing effect of the AM beneath the gingival margin. By the above results, it must be emphasized that this study succeeded in demonstrating the advantage of using an AM in the long term, whereas on a short-term basis, it had no added benefit.

This study was tried to do in the best possible way. However, there are a few limitations to this study. In the present study, only Miller's class I gingival recession was treated and the number of subjects were just adequate. In the future, I wish someone to take up this study with a greater number of subjects.

## Conclusion

The use of CAF in combination with AM proved to be fruitful in comparison with CAF alone. It is an effective and less invasive modality in treating Miller's class I gingival recession. This novel technique helps in the conservation of structural and anatomical contour of regenerated tissues. A similar root coverage could be obtained by using any of the methods, but it can be retained for a longer time by the use of the AM. This technique resulted in attaining a greater WKG. Hence, it is advisable to use AM in the treatment of class I Miller's recession for long-term stable results.

Gingival recession creates an esthetic problem, especially in the anterior region of the oral cavity. It is also associated with the dentinal hypersensitivity. Currently, there are no surgical technique or biomaterials to obtain complete gingival coverage [29]. Hence, newer biomaterials can be tried with existing surgical techniques to achieve maximum coverage. In this study, we have used AM, which is a relatively more modern biomaterial for gingival coverage showing a promising result [60]. To the best of the author's knowledge, this is the only long-term study with AM in gingival recession. Hence, further studies are needed to prove the efficacy of the amniotic membrane.

## Data Availability Statement

The datasets generated for this study are available on request to the corresponding author.

## Ethics Statement

The studies involving human participants were reviewed and approved by Karnavati School of Dentistry, Karnavati University. The patients/participants provided their written informed consent to participate in this study.

## Author Contributions

SK: conception of the idea about this study and proofreading of the prepared manuscript. TH and SB: conduction of the surgical procedure on the patients. SS: collection and analysis of data. RM: preparation of the manuscript. DS: conduction of the surgical procedure and provision of postoperative care to all the subjects. All authors contributed to the article and approved the submitted version.

## Conflict of Interest

The authors declare that the research was conducted in the absence of any commercial or financial relationships that could be construed as a potential conflict of interest.
